# Assessment of Clinical and Virological Characteristics of SARS-CoV-2 Infection Among Children Aged 0 to 4 Years and Their Household Members

**DOI:** 10.1001/jamanetworkopen.2022.27348

**Published:** 2022-08-31

**Authors:** Ruth A. Karron, Marissa K. Hetrich, Yu Bin Na, Maria Deloria Knoll, Elizabeth Schappell, Jennifer Meece, Erika Hanson, Suxiang Tong, Justin S. Lee, Vic Veguilla, Fatimah S. Dawood

**Affiliations:** 1 Department of International Health, Bloomberg School of Public Health, Johns Hopkins University, Baltimore, Maryland; 2 Marshfield Clinic Research Institute, Marshfield, Wisconsin; 3 Wisconsin State Laboratory of Hygiene, Madison; 4 COVID-19 Response, Centers for Disease Control and Prevention, Atlanta, Georgia

## Abstract

**Importance:**

Few studies have prospectively assessed SARS-CoV-2 community infection in children aged 0 to 4 years. Information about SARS-CoV-2 incidence and clinical and virological features in young children could help guide prevention and mitigation strategies.

**Objective:**

To assess SARS-CoV-2 incidence, clinical and virological features, and symptoms in a prospective household cohort and to compare viral load by age group, symptoms, and SARS-CoV-2 lineage in young children, older children, and adults.

**Design, Setting, and Participants:**

This prospective cohort study enrolled 690 participants from 175 Maryland households with 1 or more children aged 0 to 4 years between November 24, 2020, and October 15, 2021. For 8 months after enrollment, participants completed weekly symptom questionnaires and submitted self-collected nasal swabs for SARS-CoV-2 qualitative real-time reverse transcriptase polymerase chain reaction (RT-PCR) testing, quantitative RT-PCR testing, and viral lineage determination. For the analyses, SARS-CoV-2 Alpha and Delta lineages were considered variants of interest or concern. Sera collected at enrollment and at approximately 4 months and 8 months after enrollment were assayed for SARS-CoV-2 spike and nucleocapsid protein antibodies.

**Main Outcomes and Measures:**

Incidence, clinical and virological characteristics, and symptoms of SARS-CoV-2 infection by age group and correlations between (1) highest detected viral load and symptom frequency and (2) highest detected viral load and SARS-CoV-2 lineage.

**Results:**

Among 690 participants (355 [51.4%] female and 335 [48.6%] male), 256 individuals (37.1%) were children aged 0 to 4 years, 100 (14.5%) were children aged 5 to 17 years, and 334 (48.4%) were adults aged 18 to 74 years. A total of 15 participants (2.2%) were Asian, 24 (3.5%) were Black, 603 (87.4%) were White, 43 (6.2%) were multiracial, and 5 (0.7%) were of other races; 33 participants (4.8%) were Hispanic, and 657 (95.2%) were non-Hispanic. Overall, 54 participants (7.8%) had SARS-CoV-2 infection during the surveillance period, including 22 of 256 children (8.6%) aged 0 to 4 years, 11 of 100 children (11.0%) aged 5 to 17 years, and 21 of 334 adults (6.3%). Incidence rates per 1000 person-weeks were 2.25 (95% CI, 1.28-3.65) infections among children aged 0 to 4 years, 3.48 (95% CI, 1.59-6.61) infections among children aged 5 to 17 years, and 1.08 (95% CI, 0.52-1.98) infections among adults. Children aged 0 to 17 years with SARS-CoV-2 infection were more frequently asymptomatic (11 of 30 individuals [36.7%]) compared with adults (3 of 21 individuals [14.3%]), with children aged 0 to 4 years most frequently asymptomatic (7 of 19 individuals [36.8%]). The highest detected viral load did not differ between asymptomatic vs symptomatic individuals overall (median [IQR], 2.8 [1.5-3.3] log_10_ copies/mL vs 2.8 [1.8-4.4] log_10_ copies/mL) or by age group (median [IQR] for ages 0-4 years, 2.7 [2.4-4.4] log_10_ copies/mL; ages 5-17 years: 2.4 [1.1-4.0] log_10_ copies/mL; ages 18-74 years: 2.9 [1.9-4.6] log_10_ copies/mL). The number of symptoms was significantly correlated with viral load among adults (*R* = 0.69; *P* < .001) but not children (ages 0-4 years: *R* = 0.02; *P* = .91; ages 5-17 years: *R* = 0.18; *P* = .58). The highest detected viral load was greater among those with Delta variant infections (median [IQR], 4.4 [3.9-5.1] log_10_ copies/mL) than those with infections from variants not of interest or concern (median [IQR], 1.9 [1.1-3.6] log_10_ copies/mL; *P* = .009) or those with Alpha variant infections (median [IQR], 2.6 [2.3-3.4] log_10_ copies/mL; *P* = .006).

**Conclusions and Relevance:**

In this study, SARS-CoV-2 infections were frequently asymptomatic among children aged 0 to 4 years; the presence and number of symptoms did not correlate with viral load. These findings suggest that symptom screening may be insufficient to prevent outbreaks involving young children.

## Key Points

**Question:**

Do community-acquired SARS-CoV-2 infections differ in adults and children aged 0 to 4 years with respect to incidence, symptoms, and detected viral load?

**Findings:**

In this cohort study of 690 participants from 175 households in Maryland conducted from November 2020 to October 2021, 54 incident SARS-CoV-2 infections were detected in 8.6% of children aged 0 to 4 years, 11.0% of children aged 5 to 17 years, and 6.3% of adults. Children were more frequently asymptomatic or mildly symptomatic than adults; highest detected viral loads correlated with the number of symptoms in adults but not in young children.

**Meaning:**

This study’s findings suggest that symptomatic screening for SARS-CoV-2 infection may be insufficient to control outbreaks in settings in which young children congregate.

## Teaser

This cohort study assesses SARS-CoV-2 incidence, clinical and virological features, and symptoms among children aged 0 to 4 years compared with older children and adults.

## Introduction

The clinical and epidemiological characteristics of SARS-CoV-2 infection in children have been gradually elucidated during the COVID-19 pandemic.^1^ Although multiple studies have estimated incidence rates of pediatric infections and hospitalizations,^2,3^ described clinical features,^4^ and documented transmission of SARS-CoV-2 by children,^5,6,7^ most have examined children younger than 18 years collectively or have disproportionately focused on school-aged children and adolescents.^2,3,4,5,6,7,8^ Young children may differ from older children in their clinical presentation and immune response to SARS-CoV-2 and their social interaction patterns, each of which may have implications for disease burden. The burden of pediatric COVID-19 has become increasingly apparent with the Delta and Omicron variants.^9,10,11^ Children aged 0 to 4 years are the final age group for which COVID-19 vaccines have been made available in the US. Improved understanding of the epidemiological characteristics of SARS-CoV-2 infections in young children will help facilitate vaccine implementation in this age group.

The SARS-CoV-2 Epidemiology and Response in Children (SEARCH) longitudinal household cohort study was designed to examine SARS-CoV-2 epidemiological features among children aged 0 to 4 years and their household members using intensive routine weekly molecular and serological surveillance in both healthy and symptomatic individuals. The SEARCH study was conducted when SARS-CoV-2 ancestral lineages not considered variants of interest or variants of concern (non-VOI/VOC) were circulating, followed by Alpha and Delta variant lineages. This study assessed the incidence, clinical and virological characteristics, and symptoms of SARS-CoV-2 infections and examined the correlations between SARS-CoV-2 viral load and symptoms by age group and between viral load and SARS-CoV-2 lineage.

## Methods

### Household Identification, Recruitment, and Enrollment

A convenience sample of households with 1 or more child aged 0 to 4 years was enrolled in the SEARCH study from Baltimore City and selected other counties (including Anne Arundel, Baltimore, Calvert, Frederick, Harford, Howard, Montgomery, and Prince George’s) in Maryland (eMethods in Supplement 1). After enrollment, households participated in surveillance for SARS-CoV-2 infection for up to 34 weeks. Surveillance was conducted between November 24, 2020, and October 15, 2021 (eFigure 1 in Supplement 1). This study was approved by the institutional review board of Johns Hopkins University Bloomberg School of Public Health; the institutional review board of the Centers for Disease Control and Prevention approved the study based on the review of the Johns Hopkins University Bloomberg School of Public Health. Adults from eligible households provided written informed consent for themselves and for children younger than 7 years. Children provided written assent (in addition to parental consent) if they were aged 7 to 17 years. This study followed the Strengthening the Reporting of Observational Studies in Epidemiology (STROBE) reporting guideline for cohort studies.

### Data and Specimen Collection

Study questionnaires were completed electronically by adults and parents of children (with age-appropriate input from children). Enrollment questionnaires included questions about sociodemographic characteristics, social and health history, previous diagnosis of COVID-19 by a health care professional, and in-person attendance at a childcare facility, school, or work. Adults were asked about teleworking and close contact with others (<6 feet) at in-person work. For each household, a single reporter completed a questionnaire about household characteristics.

Custom-designed anterior nasal swab collection kits containing viral transport medium were provided at enrollment; instructions for collection, packaging, and shipment on cold packs were provided by televisit and video. For 8 months, participants completed weekly symptom questionnaires that asked whether they had developed fever, cough, shortness of breath or difficulty breathing, chills, sore throat, diarrhea, muscle aches, change in taste or smell, or other symptoms during the past 7 days. Participants also submitted weekly self-collected nasal swab specimens regardless of illness status; adults collected nasal swab specimens from young children. Participants collected an additional nasal swab if they developed any of the specific symptoms listed previously. Packaged nasal swabs were picked up by courier on the day of sampling and shipped overnight to the Marshfield Clinical Research Institute for performance of real-time reverse transcriptase polymerase chain reaction (RT-PCR) testing to detect SARS-CoV-2 infection.

Symptomatic participants (or parents of symptomatic children aged <18 years) completed surveys that included a longer list of respiratory and systemic symptoms.^3^ Solicited symptoms in participants 2 years and older included fever, feverishness, or chills; muscle or body aches; joint pain; change in taste or smell; headache; abnormal fatigue; nasal congestion or runny nose; sore throat; cough; shortness of breath or difficulty breathing; chest pain; abdominal pain; diarrhea; nausea or vomiting; and eye redness or rash. Solicited symptoms in children younger than 2 years included fever or feverishness, abnormal fatigue, nasal congestion or runny nose, sore throat, cough, shortness of breath or difficulty breathing, diarrhea, vomiting, eye redness or rash, and fussiness or inconsolable crying. Information on receipt of COVID-19 vaccines was solicited monthly. Serum specimens were obtained at enrollment and at approximately 4 months and 8 months after enrollment. A comprehensive summary of data elements and specimens collected is provided in eFigure 2 in Supplement 1. Data were collected and stored using Research Electronic Data Capture (REDCap) tools^12^ hosted at Johns Hopkins University.

### Antibody Assays

Sera were tested for antibodies against the nucleocapsid and spike protein receptor binding domain of wild-type SARS-CoV-2 using 2 immunoassays (Elecsys-N for the nucleocapsid protein and Elecsys-S for the spike protein; Roche Diagnostics).^13^ Both assays were highly concordant in unvaccinated individuals. Further details are provided in eMethods in Supplement 1. Results from the nucleocapsid antibody assay were used to define serologically confirmed infections because vaccination with the COVID-19 vaccines used in the US does not induce antibody to the nucleocapsid protein of SARS-CoV-2.

### Virus Detection and Genome Sequencing

Nasal swabs were tested for SARS-CoV-2 using a qualitative RT-PCR assay at the Marshfield Clinic Research Institute, as previously described.^14^ All specimens with a positive result for SARS-CoV-2 and a cycle threshold value lower than 30 were further characterized by quantitative RT-PCR (qRT-PCR) assays at the Wisconsin State Laboratory of Hygiene.^14^ The limit of detection by qRT-PCR was 1.0 log_10_ copies/mL. For assessment of the correlation between symptoms and viral load, specimens with qualitative RT-PCR cycle threshold values greater than 30 that precluded testing by qRT-PCR were assigned a value of 0.2 log_10_ copies/mL.

Specimens with cycle threshold values lower than 30 were processed for viral whole genome sequencing at the Centers for Disease Control and Prevention (eMethods in Supplement 1). For lineage identification, 1 specimen per individual per infection episode was sequenced. Specimens were classified as non-VOI/VOC (including B.1, B.1.1, B.1.2, B.1.1.434, B.1.596, and B.1.605), Alpha variant (B.1.1.7), or Delta variant (including B.1.617.2, AY.3, and AY.44) lineages.

### Analytic Definitions

We defined SARS-CoV-2 infection that occurred during the surveillance period as (1) having 1 or more nasal swab specimen with a positive result for SARS-CoV-2 during the surveillance period or (2) having SARS-CoV-2 nucleocapsid antibody seronegative results at enrollment and nucleocapsid antibody seropositive results at 4 months or 8 months after enrollment. SARS-CoV-2 infections were categorized as serologically confirmed if they met the second definition only. Fully vaccinated individuals had completed a primary COVID-19 vaccine series 2 or more weeks before the indicated time point (eTable 2 in Supplement 1). Study completion was defined as participation with fewer than 4 missed weeks of reporting and nasal swab sample collection. Participant-reported underlying medical conditions were categorized based on whether available data suggested that the condition conferred an increased risk of experiencing severe COVID-19 (eg, asthma, obesity, or overweight) (Table).

**Table.** Characteristics of SARS-CoV-2 Epidemiology and Response in Children Participants With SARS-CoV-2 Infection During Surveillance Period

Characteristic	Participants, No./total No. (%)		
	Overall	Children	Adults
Total participants, No.	54	33	21
Age category, y			
0-4	22/54 (40.7)	22/33 (66.7)	NA
<1	3/54 (5.6)	3/33 (9.1)	NA
1	3/54 (5.6)	3/33 (9.1)	NA
2	4/54 (7.4)	4/33 (12.1)	NA
3	8/54 (14.8)	8/33 (24.2)	NA
4	4/54 (7.4)	4/33 (12.1)	NA
5-17	11/54 (20.4)	11/33 (33.3)	NA
5-11	10/54 (18.5)	10/33 (30.0)	NA
12-17	1/54 (1.9)	1/33 (3.0)	NA
18-45^a^	21/54 (38.9)	NA	21/21 (100)
Sex^b^			
Female	25/54 (46.3)	14/33 (42.4)	11/21 (52.4)
Male	29/54 (53.7)	19/33 (57.6)	10/21 (47.6)
Race^b^			
Asian	3/54 (5.6)	1/33 (3.0)	2/21 (9.5)
Black	1/54 (1.9)	0	1/21 (4.8)
White	41/54 (75.9)	24/33 (72.7)	17/21 (81.0)
Multiracial	9/54 (16.7)	8/33 (24.2)	1/21 (4.8)
Ethnicity^b^			
Hispanic	1/54 (1.9)	1/33 (3.0)	0
Non-Hispanic	53/54 (98.1)	32/33 (97.0)	21/21 (100)
High risk of severe COVID-19			
Any^c^	35/54 (64.8)	16/33 (48.5)	19/21 (90.5)
Asthma	6/54 (11.1)	4/33 (12.1)	2/21 (9.5)
Obesity	17/48 (35.4)	6/27 (22.2)	11/21 (52.4)
Overweight	11/48 (22.9)	5/27 (18.5)	6/21 (28.6)
Other	3/53 (5.7)	1/32 (3.1)	2/21 (9.5)
None	19/54 (35.2)	17/33 (51.5)	2/21 (9.5)
Regularly attends activities outside the home^d^			
Yes	26/54 (48.1)	15/33 (45.5)	11/21 (52.4)
No	28/54 (51.9)	18/33 (54.5)	10/21 (47.6)
No. of household members			
2-3	16/54 (29.6)	9/33 (27.3)	7/21 (33.3)
4-5	19/54 (35.2)	11/33 (33.3)	8/21 (38.1)
≥6	19/54 (35.2)	13/33 (39.4)	6/21 (28.6)
Household income, $			
50 000 to <75 000	19/52 (36.5)	12/32 (37.5)	7/20 (35.0)
75 000 to <100 000	5/52 (9.6)	3/32 (9.4)	2/20 (10.0)
100 000 to <150 000	9/52 (17.3)	6/32 (18.8)	3/20 (15.0)
150 000 to <200 000	17/52 (32.7)	10/32 (31.3)	7/20 (35.0)
≥200 000	2/52 (3.8)	1/32 (3.1)	1/20 (5.0)
Maryland county			
Anne Arundel	20/54 (37.0)	11/33 (33.3)	9/21 (42.9)
Baltimore City	6/54 (11.1)	4/33 (12.1)	2/21 (9.5)
Baltimore County	16/54 (29.6)	11/33 (33.3)	5/21 (23.8)
Howard	1/54 (1.9)	0	1/21 (4.8)
Other^e^	11/54 (20.4)	7/33 (21.2)	4/21 (19.0)
Self-collected nasal swabs/person, median (IQR)	33 (32-35)	33 (32-35)	33 (32-35)
Sera collected, No. of specimens			
1	1/54 (1.9)	1/33 (3.0)	0
2	6/54 (11.1)	5/33 (15.2)	1/21 (4.8)
≥3^f^	47/54 (87.0)	27/33 (81.8)	20/21 (95.2)

Abbreviation: NA, not applicable.

^a^ Adults up to age 74 years were enrolled, but no individuals older than 45 years developed SARS-CoV-2 infection during the study.

^b^ Sex, race, and ethnicity were self-reported.

^c^ Includes participants who reported 1 or more of the following: cancer, chronic kidney disease, chronic lung disease, current or former smoking, diabetes, heart condition, HIV infection, immunocompromised state, liver disease, neurological conditions, overweight or obesity, pregnancy, sickle cell anemia or thalassemia, or stroke or cerebrovascular disease.^15^

^d^ Regularly attending activities outside the home was defined as childcare outside the home for participants aged 0 to 4 years, school outside the home for participants aged 5 to 17 years, and working outside the home for participants 18 years and older.

^e^ Includes Calvert, Frederick, Harford, Montgomery, and Prince George’s counties.

^f^ A fourth specimen was collected from children aged 0 to 4 years who had reverse transcriptase polymerase chain reaction–confirmed SARS-CoV-2 infection.

### Statistical Analysis

Frequencies of participant baseline characteristics and symptoms among those with SARS-CoV-2 infection were calculated. Infections were considered symptomatic if any symptom was reported during the week before the first RT-PCR positive result through 3 weeks after the last positive result; infections without reported symptoms during this period were considered asymptomatic. Incidence rates per 1000 person-weeks of RT-PCR–confirmed SARS-CoV-2 infection were calculated for the entire surveillance period and for periods of increased SARS-CoV-2 circulation, which was defined as 3 equal-length 13-week periods (period 1: November 29, 2020, to February 27, 2021; period 2: February 28, 2021, to May 29, 2021; period 3: July 18, 2021, to October 18, 2021) based on local surveillance data (eMethods in Supplement 1). SARS-CoV-2 infections were plotted by calendar week to examine the evolution of viral lineages over time.

Viral load results from qRT-PCR testing were expressed as log_10_ copies/mL. Because SARS-CoV-2 viral loads vary during infection, qRT-PCR results from specimens of symptomatic participants were excluded to allow consistent comparisons between groups. Among individuals who had 1 or more routine nasal swab specimen with qRT-PCR results available, the highest detected viral load was defined as the highest qRT-PCR value for that individual. Median highest detected viral loads were compared by SARS-CoV-2 lineage and age group. Because the highest median (IQR) detected viral load was similar in individuals with Delta variant infection who received vs did not receive a COVID-19 vaccine (median [IQR], 4.8 [3.1-5.0] log_10_ copies/mL vs 4.3 [4.1-4.9] log_10_ copies/mL), data from both groups were aggregated. A sensitivity analysis of the correlation between highest detected viral load in routine specimens and number of symptoms excluding specimens with qRT-PCR results that were assigned a value of 0.2 log_10_ copies/mL was also performed.

Fisher exact, Wilcoxon rank sum, and Spearman rank correlation tests were used as appropriate. Tests were 2-tailed, and *P* < .05 was considered statistically significant. Analyses were conducted using R software, version 4.0.3 (R Foundation for Statistical Computing), RStudio software, version 1.4.1103 (RStudio, PBC), and GraphPad Prism software, version 9.1.1 (GraphPad Software).

## Results

### Cohort Characteristics

**Figure 1.**
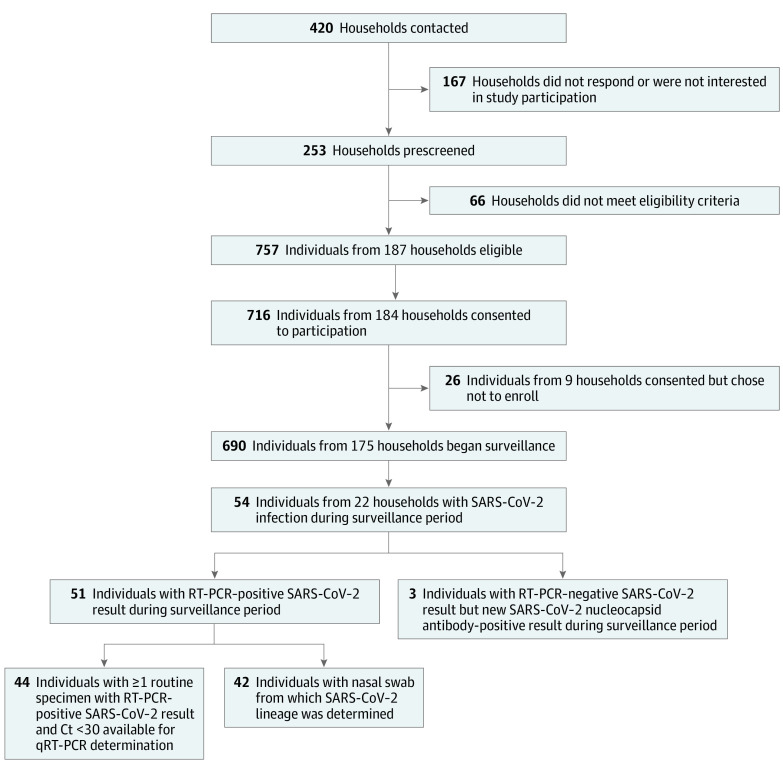
Households Contacted, Screened, and Enrolled in the SARS-CoV-2 Epidemiology and Response in Children Participants Study and Number of Participants With Evidence of SARS-CoV-2 Infection During the Surveillance Period Ct indicates cycle threshold; qRT-PCR, quantitative reverse transcriptase polymerase chain reaction; and RT-PCR, reverse transcriptase polymerase chain reaction.

From November 2020 to March 2021, 690 individuals (355 [51.4%] female and 335 [48.6%] male) from 175 households were enrolled in the SEARCH study, including 256 children (37.1%) aged 0 to 4 years (38 of whom were infants), 100 children (14.5%) aged 5 to 17 years, and 334 adults (48.4%) aged 18 to 74 years (Figure 1). A total of 15 participants (2.2%) were Asian, 24 (3.5%) were Black, 603 (87.4%) were White, 43 (6.2%) were multiracial, and 5 (0.7%) were of other races; 33 participants (4.8%) were Hispanic, and 656 (95.1%) were non-Hispanic. Most participants (361 individuals [58.2%]) resided in households with incomes of $150 000 or higher (eTable 1 in Supplement 1). At enrollment, 125 of 256 children aged 0 to 4 years (48.8%) attended childcare outside the home, and 23 of 100 children aged 5 to 17 years (23.0%) attended school in person. In total, 139 of 334 adults (41.6%) worked outside the home; of those, 113 adults (81.3%) had close (<6 feet) workplace contact with others. COVID-19 vaccine uptake was high among age-eligible participants; 307 of 335 adults (91.6%) and 15 of 18 children aged 12 to 17 years (83.3%) were fully vaccinated by study completion (eTable 2 in Supplement 1). Overall, 638 participants (92.5%) completed the study. Participants were followed up for a median (IQR) of 32 (32-33) weeks.

### SARS-CoV-2 Infections

Among 690 enrolled participants, 54 individuals (7.8%; 95% CI, 5.9%-10.1%) from 22 households had SARS-CoV-2 infection during the surveillance period, including 22 of 256 children (8.6%; 95% CI, 5.5%-12.7%) aged 0 to 4 years, 11 of 100 children (11.0%; 95% CI, 5.6%-18.8%) aged 5 to 17 years, and 21 of 334 adults (6.3%; 95% CI, 3.9%-9.5%) (Table). A total of 51 of 54 infections (94.4%) were confirmed by RT-PCR testing. Three infections in children aged 0 to 4 years were confirmed by serological results alone (Figure 1); each of those children lived with 1 or more household members who had SARS-CoV-2 infection confirmed by RT-PCR testing. Five infections occurred in fully vaccinated adults (4 received the BNT162b2 [Pfizer-BioNTech] vaccine; 1 received the mRNA-1273 [Moderna] vaccine), and 1 infection occurred in an adult who had received 1 dose of the BNT162b2 vaccine. All household members experienced SARS-CoV-2 infection in 4 of 22 households (18.2%; median [range], 5 [3-8] members per household). Two individuals were SARS-CoV-2 seropositive at enrollment but had newly positive SARS-CoV-2 PCR results on nasal swabs at 50 days and 76 days after enrollment, respectively; 1 individual was asymptomatic. Incidence rates per 1000 person-weeks of RT-PCR–confirmed SARS-CoV-2 infection were 1.84 (95% CI, 1.28-2.56) infections during the full surveillance period and 2.33 (95% CI, 1.62-3.24) infections during times of increased SARS-CoV-2 circulation. Overall, there were 2.25 (95% CI, 1.28-3.65) infections per 1000 person-weeks among children aged 0 to 4 years, 3.48 (95% CI, 1.59-6.61) infections per 1000 person-weeks among children aged 5 to 17 years, and 1.08 (95% CI, 0.52-1.98) infections per 1000 person-weeks among adults.

**Figure 2.**
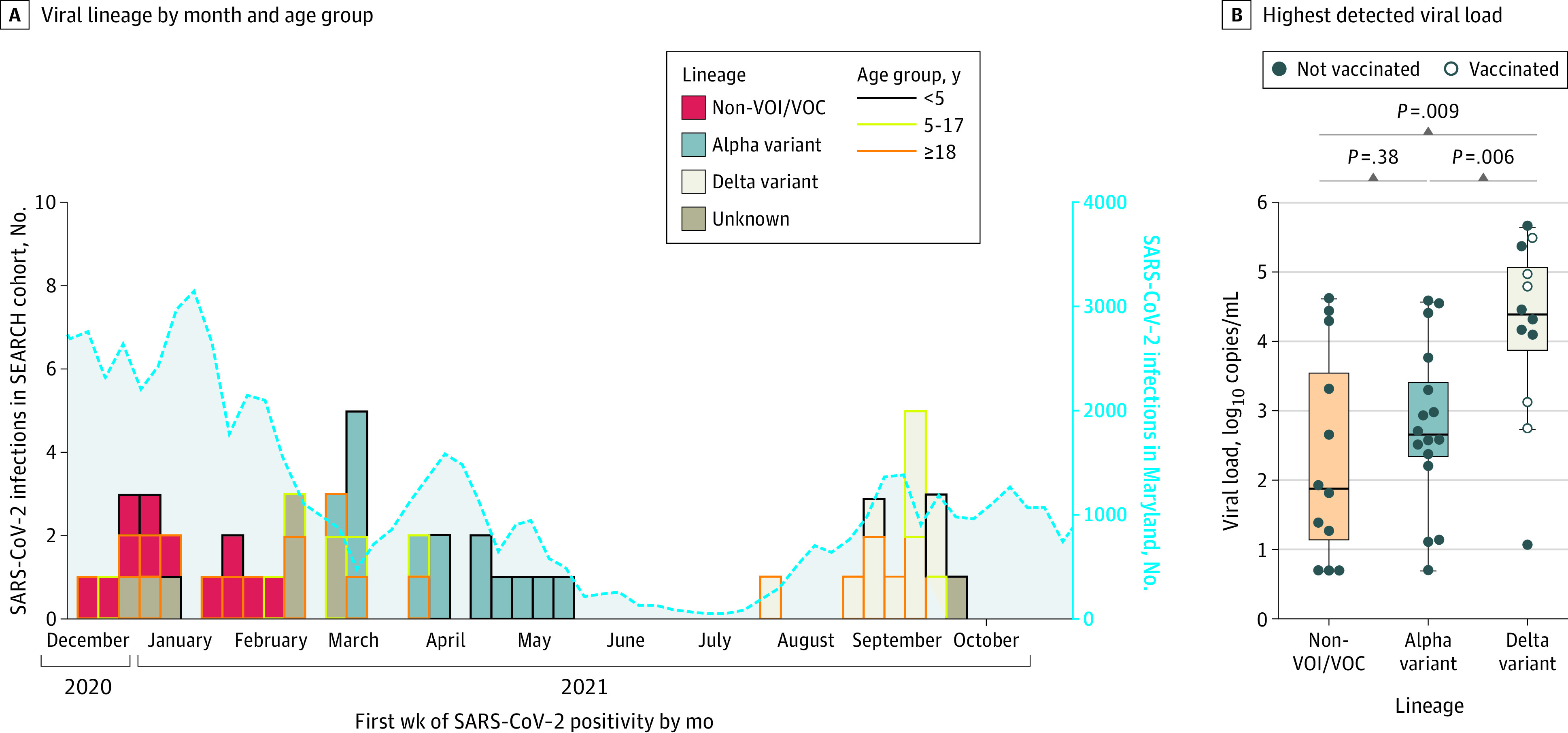
RT-PCR–Confirmed SARS-CoV-2 Infections Detected During the Surveillance Period A, Viral lineage detected by calendar month and age of participant (n = 51). Colored rules surrounding the boxes indicate age group. B, Highest detected viral load was determined by quantitative real-time reverse transcriptase polymerase chain reaction (RT-PCR) testing for non–variant of interest or variant of concern (non-VOI/VOC) ancestral lineages and Alpha and Delta variant lineages from nasal swab specimens collected at routine sampling points (n = 40). Four adults were fully vaccinated, and 1 participant was partially vaccinated before infection with the Delta variant. SEARCH indicates SARS-CoV-2 Epidemiology and Response in Children.

Of 51 participants with RT-PCR–confirmed SARS-CoV-2 infection, 42 participants (82.4%) from 21 households had specimens that were sequenced to determine viral lineage (Figure 1), including 12 individuals with non-VOI/VOC lineages, 16 with Alpha variant lineage, and 14 with Delta variant lineages. Progressive lineage displacement was observed over time (Figure 2A). Non-VOI/VOC, Alpha variant, and Delta variant lineages were detected in all age groups, although the Alpha variant was predominantly detected in children aged 0 to 4 years (of 16 individuals with infection in 8 households, 11 infections [68.8%] were among those aged 0-4 years). Five Delta variant infections occurred in fully vaccinated individuals. The highest detected viral load did not differ significantly between those with non-VOI/VOC infection (12 individuals; median [IQR], 1.9 [1.1-3.6] log_10_ copies/mL) vs Alpha variant infection (16 individuals; median [IQR], 2.6 [2.3-3.4] log_10_ copies/mL; *P* = .38) but was significantly higher among those with Delta variant infection (12 individuals; median [IQR], 4.4 [3.9-5.1] log_10_ copies/mL) vs non-VOI/VOC infection (*P* = .009) or Alpha variant infection (*P* = .006) (Figure 2B). Values among participants with Delta variant infection reflected a median highest detected viral load that was 80-fold higher than those with non-VOI/VOC infection and 25-fold higher than those with Alpha variant infection.

**Figure 3.**
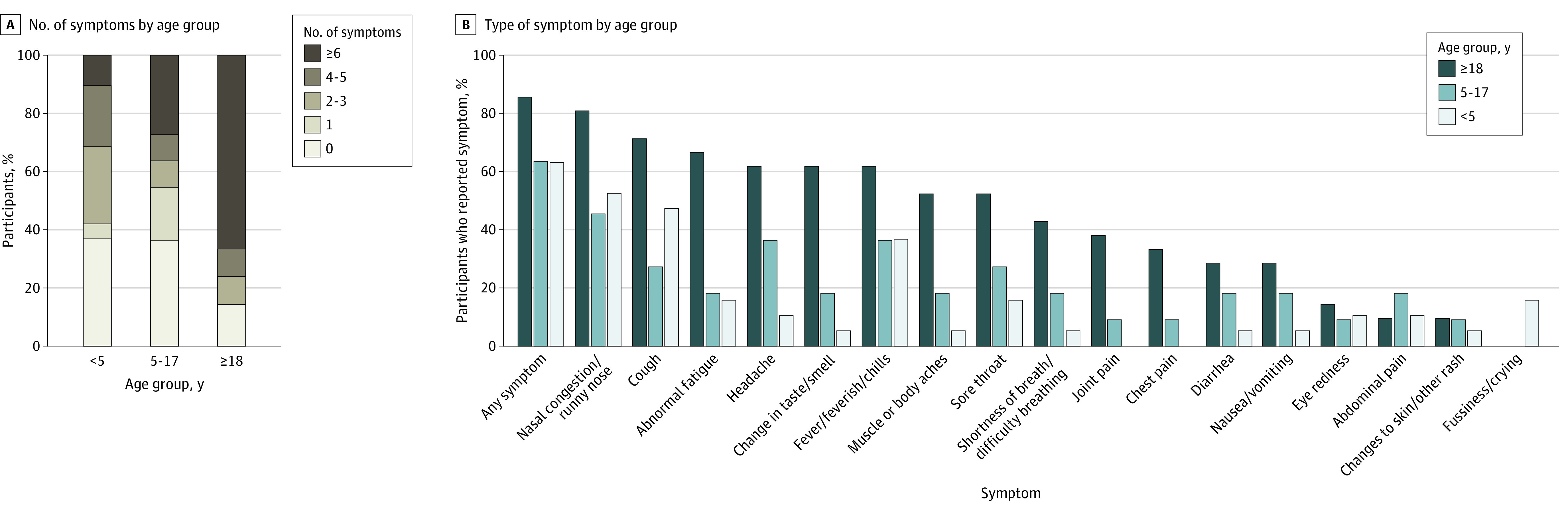
SARS-CoV-2 Symptoms by Age Group B, Solicited symptoms in participants 2 years of age and older included fever, feverishness, or chills; muscle or body aches; joint pain; change in taste or smell; headache; abnormal fatigue; nasal congestion or runny nose; sore throat; cough; shortness of breath or difficulty breathing; chest pain; abdominal pain; diarrhea; nausea or vomiting; and eye redness or rash. Solicited symptoms for children younger than 2 years included fever or feverishness, abnormal fatigue, nasal congestion or runny nose, sore throat, cough, shortness of breath or difficulty breathing, diarrhea, vomiting, eye redness or rash, and fussiness or inconsolable crying.

Among 51 participants (19 individuals aged 0-4 years, 11 aged 5-17 years, and 21 aged 18-74 years) with RT-PCR–confirmed SARS-CoV-2 infection, 14 individuals (27.5%) were asymptomatic. Children aged 0 to 17 years were more frequently asymptomatic (11 of 30 individuals [36.7%]) compared with adults (3 of 21 individuals [14.3%]), with children aged 0 to 4 years most frequently asymptomatic (7 of 19 individuals [36.8%]) (Figure 3A). Among symptomatic individuals, children aged 0 to 4 years had fewer symptoms (median [IQR], 3.5 [2.0-4.3]) than adults (median [IQR], 8.5 [6.0-11.0]; *P* = .002) and children aged 5 to 17 years (median [IQR], 5.0 [2.0-6.5]; *P* = .64). Findings were similar when the analysis was restricted to exclude symptoms that were not solicited among children younger than 2 years.

Nasal congestion was the most commonly reported symptom in all age groups (32 of 51 individuals [62.7%] with infection), whereas the relative frequency of other symptoms varied by age group. Fatigue and change in taste or smell were less common among those aged 5 to 17 years (fatigue: 2 of 11 children [18.2%]; change in taste or smell: 2 of 11 children [18.2%]) compared with adults (fatigue: 14 of 21 adults [66.7%]; *P* = .02; change in taste or smell: 13 of 21 adults [61.9%]; *P* = .03) (Figure 3B). Two participants with SARS-CoV-2 infection were hospitalized, including a child aged 2 years who had Alpha variant infection and developed multisystem inflammatory syndrome in children and a fully vaccinated adult aged 44 years with a neuromuscular disorder who had Delta variant infection and developed COVID-19 pneumonia.

**Figure 4.**
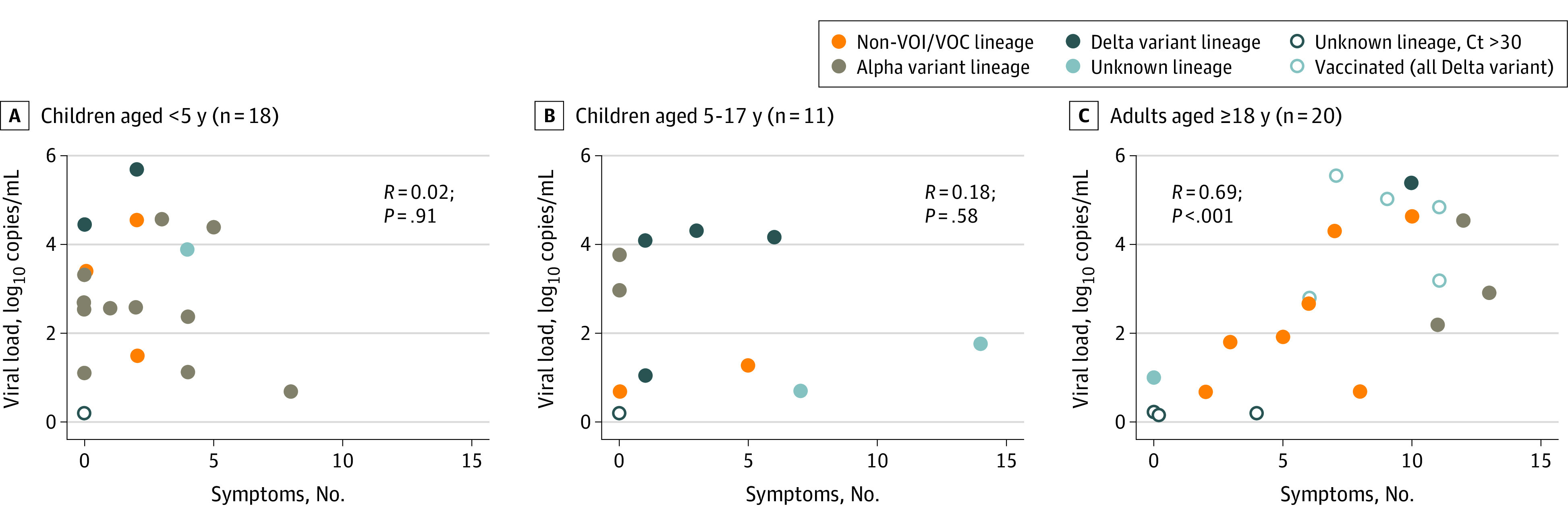
Correlation Between Number of Symptoms and Highest Detected Viral Load Four adults were fully vaccinated, and 1 participant was partially vaccinated before infection with the Delta variant. Ct indicates cycle threshold; and non-VOI/VOC, ancestral lineages not considered variants of interest or variants of concern.

Data from qRT-PCR testing of routinely collected specimens were available for 44 of 51 individuals (86.3%) with RT-PCR–confirmed infection. The highest detected viral load was similar between asymptomatic vs symptomatic individuals overall (median [IQR], 2.8 [1.5-3.3] log_10_ copies/mL vs 2.8 [1.8-4.4] log_10_ copies/mL) and by age group (median [IQR] for ages 0-4 years: 2.7 [2.4-4.4] log_10_ copies/mL; ages 5-17 years: 2.4 [1.1-4.0] log_10_ copies/mL; ages 18-74 years: 2.9 [1.9-4.6] log_10_ copies/mL). However, the correlation between median highest detected viral load and number of illness symptoms differed between adults and children. Among adults (n = 20), the median highest detected viral load was significantly correlated with the number of symptoms when qRT-PCR values and the 0.2 log_10_ copies/mL assigned value for RT-PCR–positive unquantified specimens were analyzed (*R* = 0.69; *P* < .001) (Figure 4C) and when only qRT-PCR values were analyzed (*R* = 0.53; *P* = .03) (eFigure 3 in Supplement 1). Among children aged 0 to 4 years (n = 18) and children aged 5 to 17 years (n = 11), no correlation was observed between median highest detected viral load and number of symptoms (ages 0-4 years: *R* = 0.02; *P* = .91; ages 5-17 years: *R* = 0.18; *P* = .58) (Figure 4A and B; eFigure 3 in Supplement 1). The highest detected viral loads were similar in asymptomatic vs symptomatic children (ages 0-4 years: median [IQR], 3.0 [2.6-3.3] log_10_ copies/mL vs 2.6 [1.9-4.4] log_10_ copies/mL; *P* = .96; ages 5-17 years: median [IQR], 3.0 [1.8-3.4] log_10_ copies/mL vs 1.8 [1.2-4.1] log_10_ copies/mL; *P* = .73).

## Discussion

The SEARCH cohort study was conducted during 3 SARS-CoV-2 pandemic waves in Maryland during which non-VOI/VOC ancestral lineages, the Alpha variant lineage, and Delta variant lineage of SARS-CoV-2 were sequentially predominant. A total of 7.8% of participants experienced SARS-CoV-2 infection during the surveillance period. SARS-CoV-2 infection rates were similar among children aged 0 to 4 years, children aged 5 to 17 years, and adults aged 18 to 74 years, although 91.6% of adults became fully vaccinated during the study period. SARS-CoV-2 infection was confirmed by RT-PCR testing in 51 of 54 participants with infection, supporting the use of self-collected anterior nasal swabs for sampling. Two adults with SARS-CoV-2 seropositive results at enrollment likely had reinfection, as documented by RT-PCR–confirmed SARS-CoV-2 detection during the study. No incident infections with non-VOI/VOC or Alpha variant lineages were detected in participants who were fully vaccinated against SARS-CoV-2. In contrast, infections with the Delta variant occurred in 6 vaccinated and 8 unvaccinated participants, which was consistent with observations pertaining to the risk of Delta variant infection in vaccinated populations.^16^ SARS-CoV-2 infection among all household members was rare despite the fact that all households included children aged 0 to 4 years with presumed close contact between individuals.

SARS-CoV-2 viral load may be correlated with infectiousness.^17^ In this study, we found that SARS-CoV-2 viral load and number of symptoms were highly correlated in adults, but there was no correlation between viral load and number of symptoms among children in both age groups. If viral load as measured by qRT-PCR testing is an accurate sign of infectiousness, our findings may have implications for the mitigation of SARS-CoV-2 infection, particularly among children aged 0 to 4 years. Symptom screening alone is unlikely to be sufficient to identify and stop SARS-CoV-2 outbreaks in congregate settings, such as childcare facilities. Instead, quarantine and testing of asymptomatic contacts of persons with SARS-CoV-2 infection may remain important for identifying asymptomatic individuals who might be just as likely to transmit SARS-CoV-2. Our findings also suggested that viral load in the upper respiratory tract may reflect the extent of systemic viral replication in adults but not children. Some^18,19,20^ but not all^21^ studies have found lower viral loads in asymptomatic children and adults than in symptomatic individuals. In contrast, we found no difference in viral loads between asymptomatic and symptomatic young children. The differences between our study results and previous findings may be explained by our use of quantitative (qRT-PCR testing) rather than qualitative (cycle threshold value) methods^22^ and by our intensive surveillance methods, which may have detected virus earlier in those with asymptomatic infections,^19^ when viral load may be higher.^23^

Children in both age groups who experienced RT-PCR–confirmed SARS-CoV-2 infection during the study were more frequently asymptomatic than adults. In general, children with symptomatic SARS-CoV-2 infections also experienced fewer symptoms than adults with symptomatic infections, which was consistent with previous observations.^3,18^ Although nasal congestion was frequent in all symptomatic participants,^24^ children aged 5 to 17 years were less likely than adults to experience fatigue or change in taste or smell (which could not be readily assessed in children aged <2 years). These findings highlight the challenge of identifying SARS-CoV-2 infections in young children based on symptom screening because children are more likely than adults to be asymptomatic, and SARS-CoV-2 symptoms in children are indistinguishable from symptoms associated with other common respiratory virus infections.

Viral quantification by qRT-PCR testing was performed on specimens from 46 participants; SARS-CoV-2 ancestral non-VOI/VOC, Alpha variant, and Delta variant lineages were represented. The highest detected viral load did not differ by age; however, the median highest detected viral load in individuals with Delta variant infection was 80-fold higher than that of individuals with non-VOI/VOC infection and 25-fold higher than that of individuals with Alpha variant infection. These findings were consistent with results of previous studies that have described higher viral loads in individuals with Delta variant infection.^25,26,27^ We also found that the highest detected viral load for the Delta variant did not differ in vaccinated vs unvaccinated individuals, which was consistent with findings from a study that investigated SARS-CoV-2 Delta variant dynamics.^28^

### Strengths and Limitations

This study has several strengths. We focused on infants and preschool children, a relatively understudied population with respect to SARS-CoV-2 infection. Intensive weekly surveillance in both healthy and symptomatic individuals, high adherence, and minimal attrition among cohort participants allowed for sampling throughout infection and complete symptom ascertainment. In addition, viral load was assessed using a validated quantitative PCR assay. The study also captured 3 successive pandemic waves that included circulation of SARS-CoV-2 non-VOI/VOC, Alpha, and Delta variant lineages.

The study also has limitations. As noted in a previous study,^3^ individuals who participate in intensive surveillance studies may not represent the general population, and persons of several racial and ethnic backgrounds and persons with low income were underrepresented in the SEARCH cohort; both of these issues may limit the generalizability of some findings. Moreover, the relatively small number of participants with SARS-CoV-2 infection may emphasize or minimize differences between symptomatic and asymptomatic infections. In addition, because the SEARCH study did not include individuals with Omicron variant infection, the applicability of these findings to Omicron and other newly emerging variants of concern will need to be assessed in future studies. The weekly sampling approach was not designed to identify peak viral load titers during SARS-CoV-2 infections. In addition, the quality of nasal swab specimen collection may have varied between self-collected and parent-collected specimens. However, the distribution of sample timing relative to infection onset was likely random and thus unlikely to have implications for comparisons of highest detected viral load.

## Conclusions

In this cohort study, children aged 0 to 4 years with SARS-CoV-2 infection were frequently asymptomatic or mildly symptomatic, and viral loads in their nasal swab specimens did not correlate with illness severity. Although the implications of these findings for household transmission remain to be evaluated, they suggest that SARS-CoV-2 infection may be underrecognized and that symptoms may not reflect infectiousness in young children.

## Notes

Supplement 1. **eMethods.** Recruitment, Enrollment, and Consent Procedures; Nasal Swab Specimen Shipment and Storage; Antibody Assays; Incidence Rate Calculation; Virus Sequencing; and REDCap Database **eTable 1.** Characteristics of SEARCH Participants **eTable 2.** SARS-CoV-2 Vaccine Uptake in the SEARCH Study **eFigure 1.** Number of Participants and Households Under Surveillance for SARS-CoV-2 Infection by Week, November 24, 2020, to October 15, 2021 **eFigure 2.** Unweighted Baseline Characteristics of 4587 Total Participants **eFigure 3.** Correlation Between Number of Symptoms and Highest Detected Viral Load Excluding PCR-Positive Specimens Not Evaluated by qPCR
eReferences

Supplement 2. **Nonauthor Collaborators.** SEARCH Study Team
