# Error in Discussion

**DOI:** 10.1001/jamanetworkopen.2022.35523

**Published:** 2022-09-15

In the Original Investigation titled “Assessment of Clinical and Virological Characteristics of SARS-CoV-2 Infection Among Children Aged 0 to 4 Years and Their Household Members”^1^ published August 31, 2022, a sentence in the Discussion section was incorrect. In the fifth sentence of the second paragraph, the words “quarantine and testing” should have been given as “testing,” and the full sentence should have read as follows: “Instead, testing of asymptomatic contacts of persons with SARS-CoV-2 infection may remain important for identifying asymptomatic individuals who might be just as likely to transmit SARS-CoV-2.” This article has been corrected.^1^
