# Supplementary material for: Assessment of Clinical and Virological Characteristics of SARS-CoV-2 Infection Among Children Aged 0 to 4 Years and Their Household Members
Source: JAMA Netw Open. 2022 Aug 31;5(8):e2227348. doi: 10.1001/jamanetworkopen.2022.27348 (PMC9434363; doi:10.1001/jamanetworkopen.2022.27348)
Supplement: Supplement 1. — eMethods. Recruitment, Enrollment, and Consent Procedures; Nasal Swab Specimen Shipment and Storage; Antibody Assays; Incidence Rate Calculation; Virus Sequencing; and REDCap Database eTable 1. Characteristics of SEARCH Participants eTable 2. SARS-CoV-2 Vaccine Uptake in the SEARCH Study eFigure 1. Number of Participants and Households Under Surveillance for SARS-CoV-2 Infection by Week, November 24, 2020, to October 15, 2021 eFigure 2. Unweighted Baseline Characteristics of 4587 Total Participants eFigure 3. Correlation Between Number of Symptoms and Highest Detected Viral Load Excluding PCR-Positive Specimens Not Evaluated by qPCR eReferences [file jamanetwopen-e2227348-s001.pdf]

Supplementary Online Content

Karron RA, Hetrich MK, Na YB, et al; SEARCH Study Team. Assessment of clinical and virological characteristics of SARS-CoV-2 infection among children aged 0 to 4 years and their household members. *JAMA Netw Open*. 2022;5(8):e2227348. doi:10.1001/jamanetworkopen.2022.27348

eMethods. Recruitment, Enrollment, and Consent Procedures; Nasal Swab Specimen Shipment and Storage; Antibody Assays; Incidence Rate Calculation; Virus Sequencing; and REDCap Database

eTable 1. Characteristics of SEARCH Participants

eTable 2. SARS-CoV-2 Vaccine Uptake in the SEARCH Study

eFigure 1. Number of Participants and Households Under Surveillance for SARS-CoV-2 Infection by Week, November 24, 2020, to October 15, 2021

eFigure 2. Unweighted Baseline Characteristics of 4587 Total Participants

eFigure 3. Correlation Between Number of Symptoms and Highest Detected Viral Load Excluding PCR-Positive Specimens Not Evaluated by qPCR

eReferences

This supplementary material has been provided by the authors to give readers additional information about their work.

© 2022 Karron RA et al. *JAMA Network Open*.

eMethods. Recruitment, Enrollment, and Consent Procedures; Nasal Swab Specimen Shipment and Storage; Antibody Assays; Incidence Rate Calculation; Virus Sequencing; and REDCap Database

Recruitment, enrollment, and consent procedures

SEARCh households were enrolled from Baltimore City and from Anne Arundel, Baltimore, Calvert, Frederick, Harford, Howard, Montgomery, and Prince George's Counties. Households were recruited by participating primary care pediatric practices via social media postings and email, and by word of mouth from study participants. Consent was acquired and documented by assigned clinical team members. Adults from eligible households provided consent for themselves and for children aged <7 years. Children provided assent if they were ages 7-17 years.

Nasal swab specimen shipment and storage

Nasal swab collection supplies were provided to participants at enrollment and stored at room temperature until used. Participants were given ice packs for specimen shipment which they stored in their freezers. Upon specimen collection, participants placed their samples in an insulated packing envelope with a frozen ice pack and placed them inside a biospecimen containment box. The box was stored in personal refrigerators pending pick-up by a commercial shipping company which transported the specimens overnight to the testing facility.

Antibody assays

Elecsys-N and Elecsys-S immunoassays were performed on all study sera. Elecsys-N and Elecsys-S results were highly concordant at enrollment (55 of 56 N+ and S+, 1 N- S+), and N antibodies persisted over time (53/53 sera still positive at 4 months, and 49/50 still positive at 8 months), as did S antibodies after excluding those post-vaccination (32/32 sera still positive at 4 months, and 22/22 still positive at 8 months).

Incidence rate calculations

Overall SARS-CoV-2 infection incidence rates were calculated for the full cohort period and periods of high circulation in Maryland as the number of incident RT-PCR-confirmed SARS-CoV-2 infections divided by the total number of person-weeks that participants submitted nasal swab specimens. High circulation periods were defined as three equal-length (13-week) time periods based on local surveillance data (https://covid.cdc.gov/covid-data-

© 2022 Karron RA et al. *JAMA Network Open*.

tracker/#datatracker-home): Period 1: November 29, 2020 – February 27, 2021; Period 2: February 28, 2021 – May 29, 2021; Period 3: July 18, 2021 – October 18, 2021. For incidence rate calculations, individuals who contributed ≥1 surveillance nasal swab were included, and individuals who were SARS-CoV-2 seropositive or PCR+ at baseline were excluded. Person-time was censored after the week of an individual's first positive nasal swab. Person-time after receipt of COVID-19 vaccination was included because SARS-CoV-2 infections after COVID-19 vaccination were observed in the cohort.

Virus sequencing

Specimens with Ct values <30 were processed for whole genome sequencing at the Centers for Disease Control and Prevention by previously published methods [1,2] or using the IDT xGen SARS-CoV-2 library prep kit (Integrated DNA Technologies, Inc., Coralville, IA). Libraries were sequenced using 2x150 base pair Illumina Chemistry on a MiSeq or NovaSeq instrument (Illumina Inc., San Diego, CA). Demultiplexed data were down-sampled to 1 million reads per sample, primers were trimmed with BBDuk (BBMap v38.87; sourceforge.net/projects/bbmap/), and a single consensus genome for each sample was generated with IRMA v1.0.2 using the default CoV configuration. We performed clade assignments using Nextclade version 1.13.2 [3] and assigned lineages using Pangolin version 3.1.20 [4] [pangoLEARN 1.2.123, Scorpio 0.3.16].

REDCap database

Study data were collected and managed using REDCap electronic data capture tools hosted at Vanderbilt University Medical Center with grant support UL1 TR000445 from NCATS/NIH. REDCap (Research Electronic Data Capture) is a secure, web-based software platform designed to support data capture for research studies, providing 1) an intuitive interface for validated data capture; 2) audit trails for tracking data manipulation and export procedures; 3) automated export procedures for seamless data downloads to common statistical packages; and 4) procedures for data integration and interoperability with external sources.

© 2022 Karron RA et al. *JAMA Network Open*.

eTable 1. Characteristics of SEARCH Participants

	No. (%)		
**Characteristic**	**Overall (N = 690)**	**Children (N = 356)**	**Adults (N = 334)**
**Age category**			
0-4 years	256 (37.1)	256 (71.9)	–
<1 year	38 (5.5)	38 (10.7)	–
1 year	41 (5.9)	41 (11.5)	–
2 years	45 (6.5)	45 (12.6)	–
3 years	71 (10.3)	71 (19.9)	–
4 years	61 (8.8)	61 (17.1)	–
5-17 years	100 (14.5)	100 (28.1)	–
5-11 years	85 (12.3)	85 (23.9)	–
12-17 years	15 (2.2)	15 (4.2)	–
≥18 years	334 (48.4)	–	334 (100.0)
18-49 years	318 (46.1)	–	318 (95.2)
50-64 years	14 (2.0)	–	14 (4.2)
≥65 years	2 (0.3)	–	2 (0.6)
**Gender**			
Female	355 (51.4)	177 (49.7)	178 (53.3)
Male	335 (48.6)	179 (50.3)	156 (46.7)
**Self-reported Race**			
Asian	15 (2.2)	5 (1.4)	10 (3.0)
Black	24 (3.5)	11 (3.1)	13 (3.9)
Multiracial	43 (6.2)	34 (9.6)	9 (2.7)
White	603 (87.4)	306 (86.0)	297 (88.9)
Other	5 (0.7)	0 (0.0)	5 (1.5)
**Ethnicity**			
Hispanic	33 (4.8)	19 (5.3)	14 (4.2)
Non-Hispanic	657 (95.2)	337 (94.7)	320 (95.8)
**High risk for severe COVID-19[a]**	367 (53.2)	111 (31.2)	256 (76.6)
Asthma	94 (13.6)	33 (9.3)	61 (18.3)
Overweight	164 (28.5)	45 (17.9)	119 (36.7)
Obese	125 (21.7)	35 (13.9)	90 (27.8)
Other	26 (3.8)	2 (0.6)	24 (7.2)
None	323 (46.8)	245 (68.8)	78 (23.4)
**Regularly attends activities outside the home[b]**			
Yes	287 (41.6)	148 (41.6)	139 (41.6)
No	403 (58.4)	208 (58.4)	195 (58.4)
**Household members**			
2 to 3 members	132 (19.1)	50 (14.0)	82 (24.6)
4 to 5 members	460 (66.7)	246 (69.1)	214 (64.1)
≥6 members	98 (14.2)	60 (16.9)	38 (11.4)
**Household income**			
$50,000 to less than $75,000	48 (7.7)	29 (9.1)	19 (6.3)
$75,000 to less than $100,000	34 (5.5)	18 (5.6)	16 (5.3)
$100,000 to less than $150,000	177 (28.5)	92 (28.8)	85 (28.2)
$150,000 to less than $200,000	160 (25.8)	80 (25.1)	80 (26.6)
$200,000 or more	201 (32.4)	100 (31.3)	101 (33.6)
**County**			
Anne Arundel County	295 (42.8)	150 (42.1)	145 (43.4)
Baltimore City	119 (17.2)	59 (16.6)	60 (18.0)

© 2022 Karron RA et al. *JAMA Network Open*.

	No. (%)		
**Characteristic**	**Overall (N = 690)**	**Children (N = 356)**	**Adults (N = 334)**
Baltimore County	83 (12.0)	45 (12.6)	38 (11.4)
Howard County	127 (18.4)	68 (19.1)	59 (17.7)
Other Maryland counties	66 (9.6)	34 (9.6)	32 (9.6)
**Self-collected nasal swabs per person median (IQR)**	32 (32-33)	32 (32-33)	32 (31.3-33)
**Sera collected**			
One specimen	25 (3.6)	16 (4.5)	9 (2.7)
Two specimens	37 (5.4)	28 (7.9)	9 (2.7)
Three or more specimens[c]	625 (91.0)	310 (87.6)	315 (94.6)

[a]Participants who reported one or more of the following: overweight or obesity, cancer, chronic kidney disease, chronic lung disease, neurological conditions, diabetes, heart condition, stroke or cerebrovascular disease, HIV infection, immunocompromised state, liver disease, pregnancy, sickle cell anemia or thalassemia, or being a current or former smoker (CDC: https://www.cdc.gov/coronavirus/2019-ncov/need-extra-precautions/people-with-medical-conditions.html, Accessed Nov 11th, 2021).
[b]For participants 0-4 years of age, defined as attending daycare; for participants 5-17 years of age, defined as attending school outside the home; for participants 18 years or older, defined as working outside the home.
[c]A fourth specimen was collected from children 0-4 years with RT-PCR confirmed SARS-CoV-2 infection.

© 2022 Karron RA et al. *JAMA Network Open*.

eTable 2. SARS-CoV-2 Vaccine Uptake in the SEARCH Study[a]

Vaccine	No. (%)	
	**Children 12-17 years[b] (N=18)**	**Adults ≥18 years[b] (N=335)**
Pfizer-BioNTech	15 (83.3)	160 (47.7)
Moderna	0 (0.0)	129 (38.5)
Janssen	0 (0.0)	18 (5.4)
Not vaccinated	3 (16.7)	28 (8.4)

[a]Vaccine uptake defined as completion of two-dose schedule for Pfizer-BioNTech and Moderna vaccines and one-dose schedule of the Janssen vaccine.
[b]Age at end of study

© 2022 Karron RA et al. *JAMA Network Open*.

eFigure 1. Number of Participants and Households Under Surveillance for SARS-CoV-2 Infection by Week, November 24, 2020, to October 15, 2021

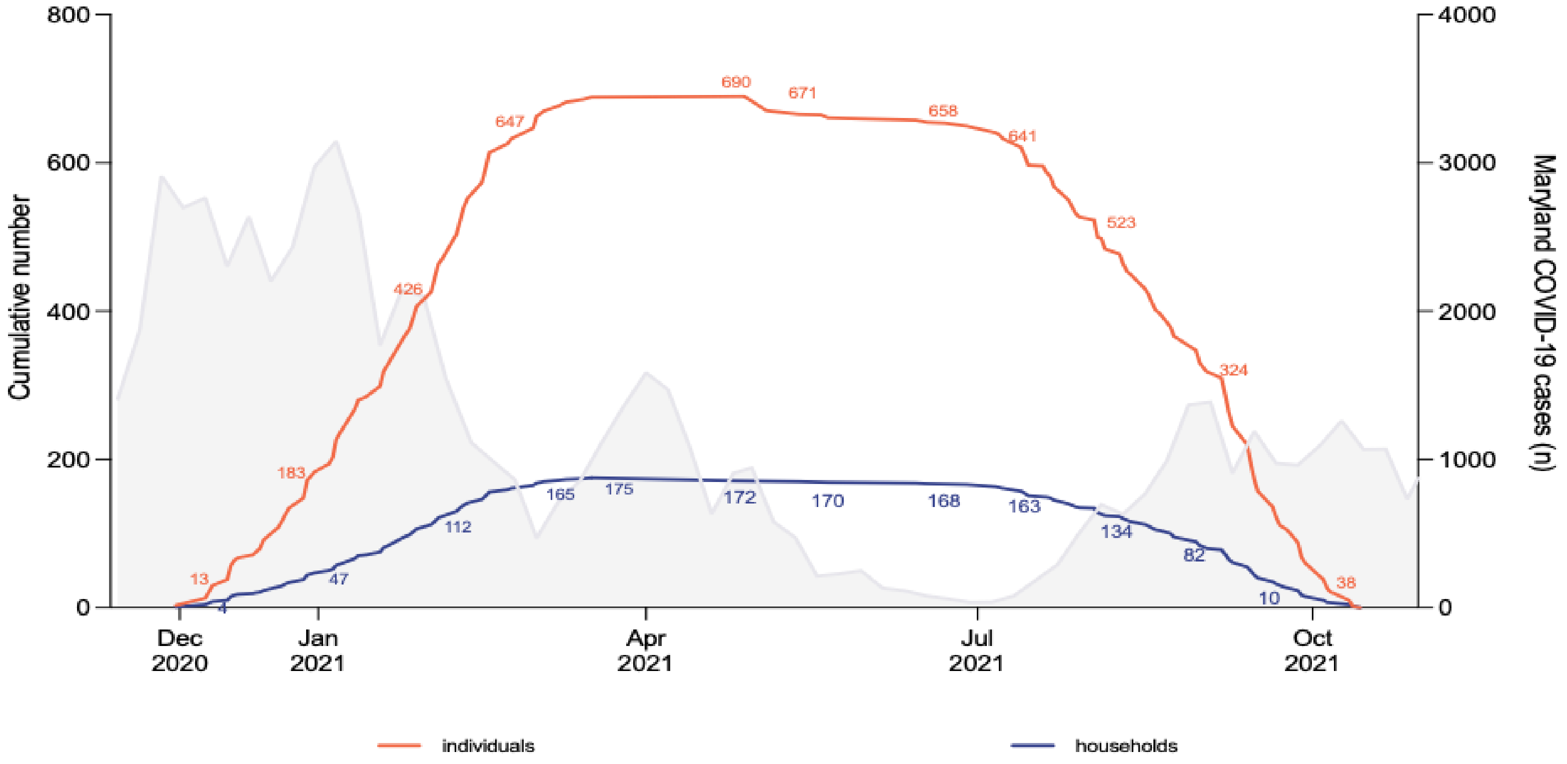

Orange lines represent total numbers of participants land black lines represent total numbers of households enrolled in the SEARCh study. Gray shading represents total number of COVID-19 cases in Maryland as reported in https://covid.cdc.gov/covid-data-tracker**.**

© 2022 Karron RA et al. *JAMA Network Open*.

eFigure 2. Information and Specimens Collected During the SEARCH Study

Data collection activity	Enrollment	Weekly (~32 weeks)	Monthly	4 months	8 months (study end)	Illness episode	4-8 weeks after PCR+
**Questionnaire**					✔		
Sociodemographic data[a]	✔						
Knowledge, attitudes, practices re SARS-CoV-2	✔				✔		
SARS-CoV-2 risk factor assessment[b]	✔	✔					
Prior history of SARS-CoV-2 infection	✔						
Psychosocial assessment of pandemic impact[c]	✔				✔		
Educational opportunities and economic impact[d]					✔		
Symptoms consistent with SARS-CoV-2 infection[e]		✔	✔		✔	✔	
Receipt of COVID-19 vaccine	✔	✔	✔		✔		
**Viral detection, quantification, & genotyping** (nasal swab)	✔	✔			✔	✔	
**SARS-CoV-2 antibodies**[f] (serum)	✔			✔	✔		(✔)

KAP = Knowledge, attitudes and practices
[a]Information collected on household size, employment, health status and healthcare coverage.
[b]Information on work, schooling, childcare, and other activities attended outside the home, and non-household members in the home.
[c]Questionnaire assessed impact of the pandemic on individual anxiety and stressors.
[d]Questionnaire assessed impact of the pandemic on housing, utilities, food, healthcare, and access to technology.
[e]Fever, cough, shortness of breath or difficulty breathing, chills, sore throat, diarrhea, muscle aches, change in taste/smell, or other symptoms during the past 7 days.
[f]Sera collected for SARS-CoV-2 antibody assays. Specimens were collected from all subjects at enrollment, and at 4 and 8 months after enrollment. Sera were also collected from children ages 0-4 years within 4-8 weeks following a SARS-CoV-2 PCR+ nasal swab, which in some instances coincided with the 4 or 8 month specimen.

© 2022 Karron RA et al. *JAMA Network Open*.

eFigure 3. Correlation Between Number of Symptoms and Highest Detected Viral Load Excluding PCR-Positive Specimens Not Evaluated by qPCR

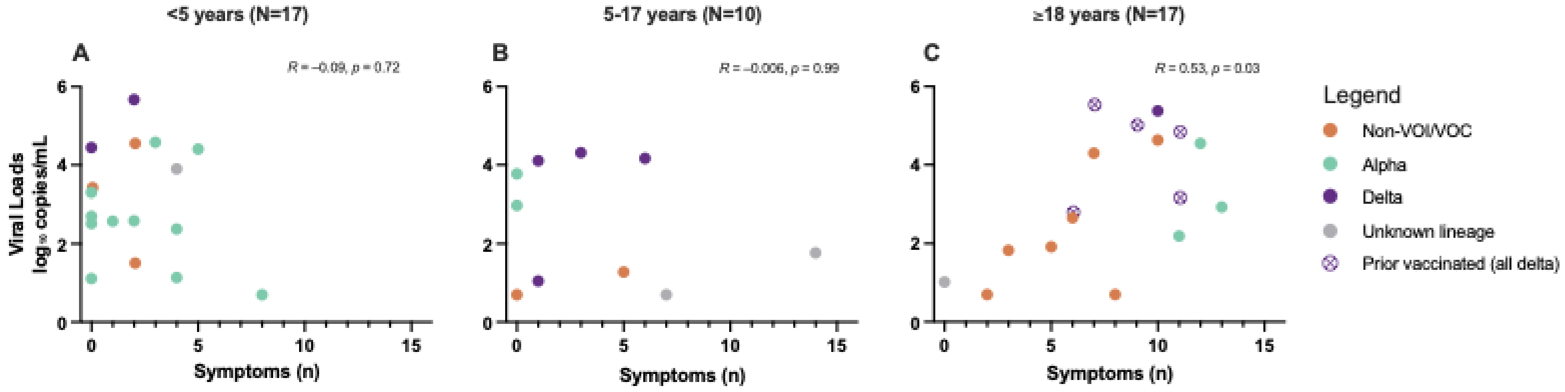

Data are shown for young children, (n=17) (**S3A**), older children (n=10) (**S3B**), and adults (n=17) (**S3C**). Number of symptoms is shown on the x axis and highest detected viral load, expressed as $\log_{10}$ copies/mL, is shown on the y axis. Four individuals were fully vaccinated prior to infection and one was partially vaccinated.

© 2022 Karron RA et al. *JAMA Network Open*.

eReferences

1. Paden, C.R., et al., *Rapid, Sensitive, Full-Genome Sequencing of Severe Acute Respiratory Syndrome Coronavirus 2.* Emerg Infect Dis, 2020. **26**(10): p. 2401-2405.
2. Shepard, S.S., et al., *Viral deep sequencing needs an adaptive approach: IRMA, the iterative refinement meta-assembler*. BMC Genomics, 2016. **17**(1): p. 708.
3. Aksamentov et al., N*extclade: clade assignment, mutation calling and quality control for viral genomes*. Journal of Open Source Software, 2021. **6**(67), 3773.
4. O'Toole, Á., et al., *Assignment of epidemiological lineages in an emerging pandemic using the pangolin tool.* Virus Evol, 2021. **7**(2): p. veab064.

© 2022 Karron RA et al. *JAMA Network Open*.
