# Supplementary material for: Assessment of Clinical and Virological Characteristics of SARS-CoV-2 Infection Among Children Aged 0 to 4 Years and Their Household Members
Source: JAMA Netw Open. 2022 Aug 31;5(8):e2227348. doi: 10.1001/jamanetworkopen.2022.27348 (PMC9434363; doi:10.1001/jamanetworkopen.2022.27348)
Supplement: Supplement 2. — Nonauthor Collaborators. SEARCH Study Team [file jamanetwopen-e2227348-s002.pdf]

Supplemental Online Content: Nonauthor Collaborators
*First name, last name, and suffix (if applicable) are required and will appear in PubMed.

***Group Name(s): SEARCH Study Team**							
***First Name and Middle Initial(s)**	***Last Name**	***Suffix (eg, Jr, III)**	Academic Degrees	Institution	Location (city, state/province, country)	Role or Contribution, eg, chair, principal investigator	Group (if more than 1 Group listed in the byline) and/or Subgroup (eg, Steering Committee)
Christine	Council-DiBitetto		RN	Johns Hopkins University		research nurse	SEARCh Study Team
Milena	Gatto		RN	Johns Hopkins University		research nurse	SEARCh Study Team
Maria	Garcia-Quesada		MSPH	Johns Hopkins University		data analyst	SEARCh Study Team
Tina	Ghasri		BS	Johns Hopkins University		laboratory scientist	SEARCh Study Team
Amanda	Gormley		RN	Johns Hopkins University		research nurse	SEARCh Study Team
Kyley	Guenther		BS	Wisconsin State Laboratory of Hygiene		laboratory scientist	SEARCh Study Team
Kristi	Herbert		RN, MSN	Johns Hopkins University		research nurse	SEARCh Study Team
Maria	Jordan		RN	Johns Hopkins University		research nurse	SEARCh Study Team
Karen	Loehr		RN	Johns Hopkins University		research nurse	SEARCh Study Team
Jason	Morsell		--	Johns Hopkins University		laboratory scientist	SEARCh Study Team
Jennifer	Oliva		BS	Johns Hopkins University		laboratory scientist	SEARCh Study Team
Jocelyn	San Mateo		RN, MSN	Johns Hopkins University		research nurse	SEARCh Study Team
Khadija	Smith		BS	Johns Hopkins University		laboratory scientist	SEARCh Study Team
Kimberli	Wanionek		BS	Johns Hopkins University		laboratory scientist	SEARCh Study Team
Cathleen	Weadon		RN	Johns Hopkins University		research nurse	SEARCh Study Team
Suzanne	Woods		RN, MSN	Johns Hopkins University		research nurse	SEARCh Study Team
Yan	Li		BS	Centers for Disease Control and Prevention		laboratory scientist	SEARCh Study Team
Anna	Kelleher		BS	Centers for Disease Control and Prevention		laboratory scientist	SEARCh Study Team
Anna	Uehara		BS	Centers for Disease Control and Prevention		laboratory scientist	SEARCh Study Team
Ying	Tao		BS	Centers for Disease Control and Prevention		laboratory scientist	SEARCh Study Team
Jing	Zhang		BS	Centers for Disease Control and Prevention		laboratory scientist	SEARCh Study Team

*First name, last name, and suffix (if applicable) are required and will appear in PubMed.

***First Name and Middle Initial(s)**	***Last Name**	***Suffix (eg, Jr, III)**	Academic Degrees	Institution	Location (city, state/province, country)	Role or Contribution, eg, chair, principal investigator	Group (if more than 1 Group listed in the byline) and/or Subgroup (eg, Steering Committee)
Brian	Lynch		BS	Centers for Disease Control and Prevention		laboratory scientist	SEARCh Study Team
Meghan	Bentz		BS	Centers for Disease Control and Prevention		laboratory scientist	SEARCh Study Team
Alex	Burgin		BS	Centers for Disease Control and Prevention		laboratory scientist	SEARCh Study Team
Mark	Burroughs		BS	Centers for Disease Control and Prevention		laboratory scientist	SEARCh Study Team
Morgan L.	Davis		BS	Centers for Disease Control and Prevention		laboratory scientist	SEARCh Study Team
Joseph C.	Madden		BS	Centers for Disease Control and Prevention		laboratory scientist	SEARCh Study Team
Sarah	Nobles		BS	Centers for Disease Control and Prevention		laboratory scientist	SEARCh Study Team
Jasmine	Padilla		BS	Centers for Disease Control and Prevention		laboratory scientist	SEARCh Study Team
Mili	Sheth		BS	Centers for Disease Control and Prevention		laboratory scientist	SEARCh Study Team
Dhwani	Bhatra		BS	Centers for Disease Control and Prevention		laboratory scientist	SEARCh Study Team
Jason	Caravas		BS	Centers for Disease Control and Prevention		laboratory scientist	SEARCh Study Team
Peter	Cook		BS	Centers for Disease Control and Prevention		laboratory scientist	SEARCh Study Team
Dakota	Howard		BS	Centers for Disease Control and Prevention		laboratory scientist	SEARCh Study Team
Kristine	Lacek		BS	Centers for Disease Control and Prevention		laboratory scientist	SEARCh Study Team

Supplemental Online Content: Nonauthor Collaborators
*First name, last name, and suffix (if applicable) are required and will appear in PubMed.

***First Name and Middle Initial(s)**	***Last Name**	***Suffix (eg, Jr, III)**	Academic Degrees	Institution	Location (city, state/province, country)	Role or Contribution, eg, chair, principal investigator	Group (if more than 1 Group listed in the byline) and/or Subgroup (eg, Steering Committee)
Clint	Paden		BS	Centers for Disease Control and Prevention		laboratory scientist	SEARCh Study Team
Ben	Rambo-Martin		BS	Centers for Disease Control and Prevention		laboratory scientist	SEARCh Study Team
Samuel	Shepard		BS	Centers for Disease Control and Prevention		laboratory scientist	SEARCh Study Team
